# Recent Advances in the Heterogeneous Photocatalytic Hydroxylation of Benzene to Phenol

**DOI:** 10.3390/molecules27175457

**Published:** 2022-08-25

**Authors:** Weiwei Han, Wei Xiang, Jun Shi, Yue Ji

**Affiliations:** College of Chemistry and Chemical Engineering, Xi’an Shiyou University, Xi’an 710065, China

**Keywords:** heterogeneous photocatalysts, hydroxylation, benzene, phenol

## Abstract

Phenol is an important chemical material that is widely used in industry. Currently, phenol is dominantly produced by the well-known three-step cumene process, which suffers from severe drawbacks. Therefore, developing a green, sustainable, and economical strategy for the production of phenol directly from benzene is urgently needed. In recent years, the photocatalytic hydroxylation of benzene to phenol, which is economically feasible and could be performed under mild conditions, has attracted more attention, and development of highly efficient photocatalyst would be a key issue in this field. In this review, we systematically introduce the recent achievements of photocatalytic hydroxylation of benzene to phenol from 2015 to mid-2022, and various heterogeneous photocatalysts are comprehensively reviewed, including semiconductors, polyoxometalates (POMs), graphitic carbon nitride (g-C_3_N_4_), metal–organic frameworks (MOFs), carbon materials, and some other types of photocatalysts. Much effort is focused on the physical and chemical approaches for modification of these photocatalysts. The challenges and future promising directions for further enhancing the catalytic performances in photocatalytic hydroxylation of benzene are discussed in the end.

## 1. Introduction

Phenol, as an industrially important chemical, is extensively used in the manufacture of bisphenol A, adipic acid, resins, fibers, nylon, herbicides, drugs, etc. [1,2,3] In recent years, the global demand for phenol has shown an increasing trend. In industry, more than 90% of phenol is produced from the well-known three-step cumene process [4,5], which suffers from several notable drawbacks such as harsh reaction conditions, low one-pass yield (~5%) of phenol, and the considerable amount of by-product [6]. To circumvent these shortfalls, the direct hydroxylation of benzene to phenol has emerged as an alternative strategy due to its advantages of high atom economy and an environmentally benign procedure [7,8]. Since the 1960s, great efforts have been devoted to developing new catalytic systems using oxygen [9], nitrous oxide [10], or hydrogen peroxide [11] as an oxidizing agent, however, it seems that there have been no significant breakthroughs [8,12]. Direct hydroxylation of benzene has been considered as one of the top 10 most difficult challenges in modern catalysis. Most of the developed catalytic systems require elevated temperature and/or high pressure and suffer from unsatisfactory conversion because of the proverbial low reactivity of aromatic C−H bonds [13]. Furthermore, phenol is liable to be over-oxidized due to its increased reactivity compared with benzene, which makes the selective introduction of the hydroxyl group into benzene very difficult, especially under traditional heating conditions [14]. Therefore, it is highly desirable to develop methods for the oxidation of benzene into phenol that are economically feasible and could be performed with high selectivity under mild conditions.

In recent years, many attempts at direct conversion of benzene to phenol have been performed by means of palladium membrane [15], electrochemical oxidation systems [16], nonthermal plasma systems [17], biocatalysis processes [18], photocatalysis systems, and utilization of renewable resources such as biomass [19], bio-oils [20], etc. Among these methods, photocatalysis that utilizes renewable and inexhaustible solar energy has drawn more attention. Furthermore, photocatalytic oxidation of benzene can take place under mild conditions. Therefore, it has been commonly recognized as a green, sustainable, and economical strategy for the production of phenol [21]. How to design and fabricate a highly selective and efficient photocatalyst is considered to be the key issue in this photocatalytic reaction.

**Figure 1:**
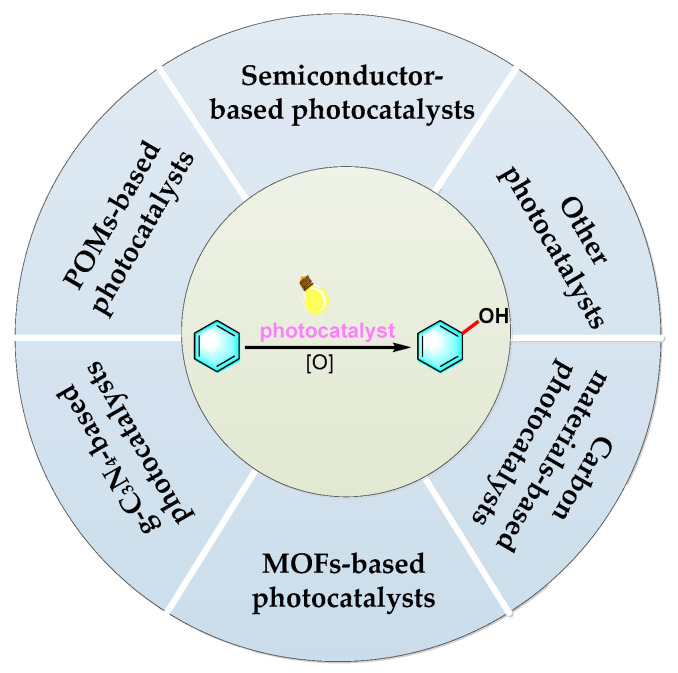
Various types of heterogeneous photocatalysts developed for hydroxylation of benzene to phenol.

In the past few years, extensive studies have been carried out on the development of various photocatalytic systems for the selective oxidation of benzene to phenol, some of which have been reviewed in previous publications [22,23,24]. Fukuzumi and coworkers [24,25,26,27,28] studied a series of homogeneous photocatalysts, such as 2,3-dichloro-5,6-dicyano-*p*-benzoquinone (DDQ), quinolines, and transition metal complexes, which exhibited excellent performance in photocatalytic phenol synthesis and the inhibition of phenol’s over-oxidation. However, there are some concerns with regard to the separation, recovery, and reusability of catalysts. In comparison with these homogeneous photocatalysts, the heterogeneous ones are cheaper to fabricate and can be easily separated for reuse, therefore, displaying promising potential in industrial application. With regard to this, various heterogeneous photocatalysts have been developed, including inorganic semiconductors, polyoxometalates (POMs), graphitic carbon nitride (g-C_3_N_4_), metal–organic frameworks (MOFs), carbon materials, and some other types of photocatalysts. This review aims to provide an updated, comprehensive review on the development of heterogeneous photocatalysts for hydroxylation of benzene to phenol, since 2015, and their catalytic performances (as depicted in Figure 1). Much attention is focused on various strategies for the modification of heterogeneous photocatalysts. In the end, the challenges and future promising directions are briefly summarized.

## 2. Heterogeneous Photocatalysts for Hydroxylation of Benzene to Phenol

Considerable efforts have been made on developing simple, efficient, and sustainable materials as heterogeneous photocatalysts over the past decades. Considering that the intrinsic characteristics of heterogeneous photocatalysts could be selectively adjusted through appropriate modifications, a variety of approaches have been explored to obtain high-efficiency heterogeneous photocatalysts for direct phenol synthesis.

### 2.1. Semiconductor-Based Photocatalysts

Photocatalysis on semiconductor materials is one of the promising candidates for the clean and direct synthesis of phenol from benzene. Among the various wide-band-gap semiconductor photocatalysts, TiO_2_ is the most extensively explored semiconductor in photocatalytic phenol synthesis due to its low cost, non-toxicity, and extraordinary chemical stability against photocorrosion. In addition, holes with high oxidizing power are generated in their valence band upon light absorption [29,30]. However, due to the wide band gap of TiO_2_, it can be activated only under ultraviolet (UV) light conditions. Unfortunately, only a small portion of solar irradiation falls in this range. In consequence, it is an attractive challenge for TiO_2_ to absorb more solar irradiation.

**Figure 2:**
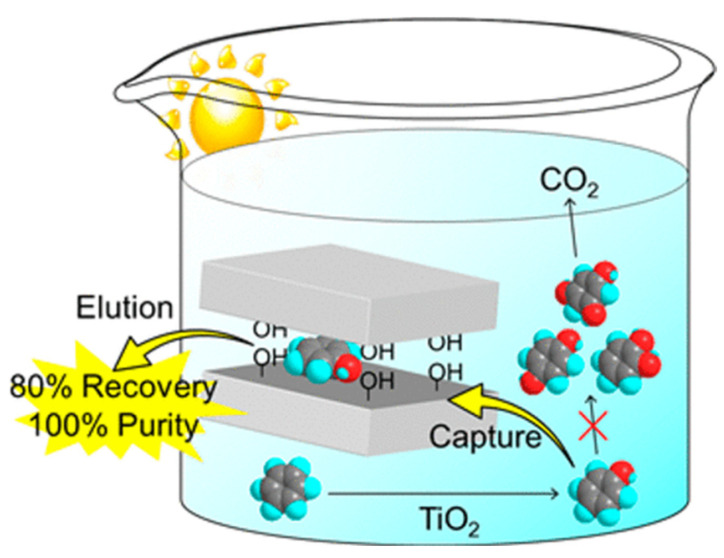
Schematic illustration of selective recovery of phenol by layered silicic acid in photocatalytic hydroxylation of benzene in water. Reprinted with permission from Ref. [33]. 2013 American Chemical Society.

In attempting to extend the photoresponse of TiO_2_ into the visible wavelength region, tris(2,2′-bipyridine) ruthenium(II) complex ([Ru(bpy)_3_]^2+^) was widely used for TiO_2_ modification. In a study by Goto et al. [31], a sodium-type synthetic saponite (SSA) was employed to separate [Ru(bpy)_3_]^2+^ from TiO_2_, while maintaining the interaction between the photo-excited complex and TiO_2_, which realized the photocatalytic benzene oxidation with the catalyst in the flow reactor. Shiraishi et al. [32] prepared disordered mesoporous TiO_2_ (mTiO_2_) with different pore sizes and specific surface areas and compared the catalytic performances of mTiO_2_ and non-porous TiO_2_ (nTiO_2_) for the hydroxylation of benzene under UV light. It was found that the phenol selectivity (81%) obtained from mTiO_2_ was 10 times more than that obtained from nTiO_2_. One potential explanation offered by the researchers is that the adsorption ability of benzene on mTiO_2_ was much stronger than that of phenol, and the rapid desorption of phenol inhibited its further oxidation on catalyst surface, resulting in higher phenol selectivity. In a report from Ide et al. [33], H_2_Si_14_O_29_ (H-mag) was used to selectively adsorb the newly formed phenol to prevent it from over-oxidizing (Figure 2). In the presence of H-mag, phenol was recycled with a high selectivity up to 100%, although the same benzene conversion was obtained.

**Figure 3:**
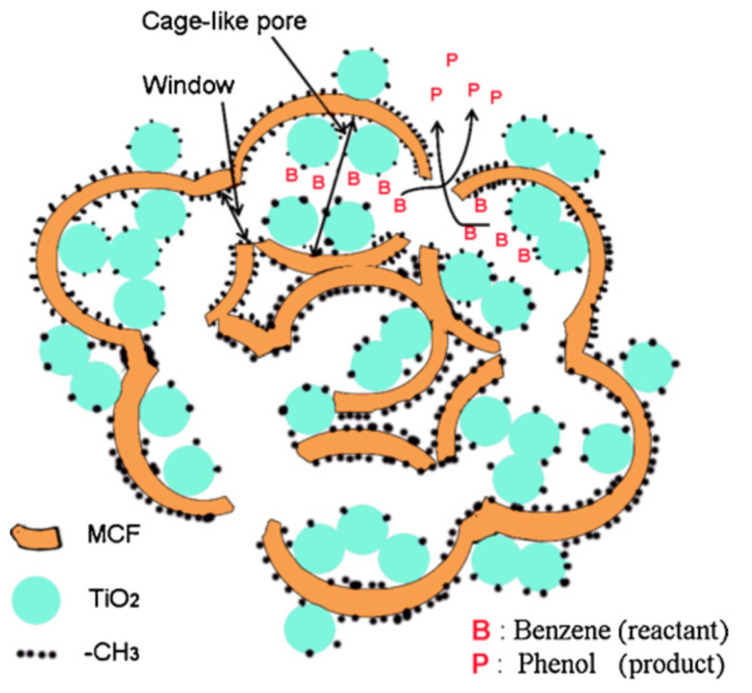
TiO_2_ entrapped in the cagelike mesopores of hydrophobically modified MCF for the hydroxylation of benzene. Reprinted with permission from Ref. [34]. 2011 Elsevier.

In the study by Zhang et al. [34], TiO_2_ nanoparticles were entrapped into a hydrophobically modified mesocellular siliceous foam (MCF) to provide a hydrophobic environment, in which the generated hydrophilic phenol could be rapidly released out of the catalyst, as schematically illustrated in Figure 3. Both the adsorption of benzene and desorption of phenol on the surface of TiO_2_ were facilitated, thereby increasing phenol selectivity from 15.8% to 34.7%.

In the past decades, hydroxylation of benzene to phenol over Au-deposited TiO_2_ (Au/TiO_2_) has been systematically studied. The excited electrons are stored in Au nanoparticles, which promote the oxidation of benzene by TiO_2_. Ide et al. [35] employed layered TiO_2_-supported Au nanoparticles for benzene oxidation under visible light. In their study, Au nanoparticles were deposited in the interlayer space of a layered titanate to form a clay-type material with molecular sieving ability, which expedited the separation of the desired product. The presence of seed phenol was demonstrated to play a pivotal role in the reaction; with the addition of an excess of seed phenol (18,000 ppm), a phenol yield of 62% can be realized, and no phenol formation was observed in the absence of seed phenol. Additionally, they also reported a facile method to improve the efficiency and selectivity of photocatalytic hydroxylation of benzene, by introducing CO_2_ into the reaction [36]. The presence of CO_2_ (230 kPa) was revealed to be able to promote the desorption of phenol, thus inhibiting the over-oxidation of phenol. In another work by Marino et al. [37], Au nanoparticles were loaded on anatase TiO_2_ for the photocatalytic benzene oxidation reaction. Compared with the reaction rate with unmodified TiO_2_, the reaction rate with Au/TiO_2_ was lower at the initial stage, but then surpassed that of TiO_2_ as the reaction time prolonged. The highest phenol yield was obtained at 1.0 wt% Au loading. Zheng et al. [38] prepared M@TiO_2_ (M: Au, Pt, Ag) composites through an efficient in situ method. In their research, TiO_2_ powder was dispersed in absolute ethanol to generate Ti^3+^ ions on the surface of TiO_2_ particles upon irradiation with UV light. The generated Ti^3+^ ions then reduced the noble-metal ions in dark, which made the noble-metal nanoparticles uniformly deposited on the surface of TiO_2_. Among these three composites, Au@TiO_2_ exhibited the highest phenol yield (63%) and selectivity (91%).

Besides the above studies on noble-metal nanoparticles and TiO_2_, Su et al. [39] investigated the selective oxidation of benzene to phenol over a series of metal nanoparticles loaded on TiO_2_, including metal (Au, Pd), alloy (Au-Pd), and shell-core (Au-Pd and Pd-Au). It was revealed that by adjusting the morphology and composition of Au-Pd bimetallic nanoparticles, the degradation of benzene and the successive oxidation of phenol could be reduced. Among these catalysts, the TiO_2_-supported Au-Pd (shell and core, respectively) nanoparticles that could simultaneously increase phenol formation rate and decrease the generation of hydroquinone exhibited the highest photocatalytic performance. Although the conversion of benzene was still low (30%), it provided a novel method for the direct synthesis of phenol with high purity. For the purpose of enhancing the trapping of excited electrons, Devaraji and coworkers [40] firstly doped V into the TiO_2_ lattice to generate a V^5+^ energy level below the conduction band (CB) of TiO_2_, followed by depositing Au on the resultant Ti_0.98_V_0.02_O_2_ (TV2), in which Au acted as an electron sink for promoting the separation and migration of electrons and holes to the catalyst surface. The Schottky junction between Au and TiO_2_ as well as V^5+^ synergistically increased the availability of holes in the valence band (VB) of TiO_2_, thereby enhancing the conversion of benzene to phenol under UV light. Considering the high thermal stability and large oxygen storage capacity of CeO_2_, dispersed Pd nanoclusters incorporated on CeO_2_/TiO_2_ nanocomposite were also developed by Ma and coworkers for selective benzene oxidation [41], in which the synergistic effect between Pd and the support was regarded as an important factor for the improved photocatalytic activity. Furthermore, the prepared catalyst could be used five times without any loss in activity. Park et al. [42] investigated the effects of TiO_2_ surface modification (platinum deposition, fluorination, SiO_2_ loading) and the combination with polyoxometalate (i.e., TiO_2_ and POM system) on photocatalytic hydroxylation of benzene. Platinum deposition and surface fluorination of TiO_2_ were thought to contribute to the generation of free •OH radicals on the catalyst surface, which could significantly improve the yield and selectivity of phenol, while the loading of SiO_2_ had little effect on the catalytic activity. When POM was added into the catalytic system, it acted as both a homogeneous photocatalyst and a reversible electron acceptor, thus increasing the phenol yield to 11%. This work offered a new way in the design of novel photocatalysts for benzene hydroxylation.

In attempting to overcome the high cost of the noble metals (e.g., Au, Pd, Pt, and Ag), numerous efforts have been explored to replace them with inexpensive materials for TiO_2_ modification. Devaraji et al. [43] prepared disordered mesoporous Ti_0.98_Fe_0.01_Cr_0.01_O_2_ by doping Fe and Cr into TiO_2_, which enabled the CB to generate an electron-trapping level. Besides, with a short carrier-diffusion length, the as-prepared co-doped TiO_2_ could inhibit the recombination of photogenerated carriers and accelerate their migration to the catalyst surface, thus resulting in enhanced UV photocatalytic activity. Cu nanoparticles have also been demonstrated as appropriate alternatives to expensive noble metals in recent studies. Tanarungsun et al. [44] investigated multiple transition metals (FeCu, FeV, and FeVCu) supported on TiO_2_ for the liquid-phase photocatalytic hydroxylation of benzene to phenol under UV light. Compared with bimetallic composites (FeCu/TiO_2_, FeV/TiO_2_), the synergistic catalytic effect observed in trimetallic composites (FeVCu/TiO_2_) facilitated benzene oxidation. In a recent work by Devaraji et al. [45], Cu(OH)_2_-loaded 2D leaf-structured dual-phase (anatase-rutile) mesoporous leaf titania (LT) was developed and exhibited enhanced photocatalytic activity in benzene hydroxylation. It was revealed that the synergistic effect of the surface–phase junction, the disordered mesoporous 2D leaf structure, and the integration of Cu^2+^ into LT had increased the mobility of the photoexcited charge carriers, thereby facilitating the benzene-to-phenol photocatalytic conversion.

Additionally, WO_3_, as an n-type semiconductor with a direct band-gap excitation at 2.4~2.8 eV, is another well-researched semiconductor for the photocatalytic synthesis of phenol from benzene [46]. However, the lower CB position of WO_3_ inhibits the generation of VB holes with higher oxidation ability. Furthermore, the high recombination of the photo-generated charges in WO_3_ would lead to a relatively low photoelectric catalytic activity [47]. Yoshida et al. [48] demonstrated that Pt-loaded TiO_2_ could significantly improve the phenol selectivity without O_2_. However, the efficiency was not ideal, possibly due to the lower capability of water (or protons) in capturing the photoexcited electrons, compared with O_2_. Thus, nano-Pt loaded on WO_3_ was employed to improve the photocatalytic performance of WO_3_ for selective oxidation of benzene in water and oxygen under UV light and visible light (300 < λ < 500 nm) [49]. The results showed that Pt/WO_3_ afforded a phenol selectivity up to 74%, which was much higher than Pt/TiO_2_ and bare TiO_2_. The mechanistic investigation demonstrated that the photoexcited electrons on the Pt/WO_3_ photocatalyst were mainly formed through the two-electron reduction of O_2_, and the generated H_2_O_2_ could not participate in the oxidation of benzene. On the contrary, the oxygen radical species generated on TiO_2_ promoted the oxidative decomposition of benzene, thus reducing the selectivity of phenol. No production of reactive oxygen radicals in O_2_ together with the ability to selectively oxidize water into •OH radicals were considered to be the reasons for the high phenol selectivity obtained from Pt/WO_3_ [50]. In Kurikawa’s recent work, the reaction mechanisms for photocatalytic hydroxylation of benzene under visible light (420 < λ < 540 nm), over Pt-WO_3_, was further investigated [51]. According to this study, Pt-WO_3_ with different contents of Pt species could absorb the light above 450 nm because of the scattering effect from the Pt particles and surface resonance, so their photocatalytic activity increased significantly with both Pt species deposited on WO_3_ and the reaction time increased. Mechanistic studies have shown that the dissociation rate of the O-H bond in water played an important role in the hydroxylation reaction, and the photogenerated H_2_O_2_ was proposed to replace the OH derived from H_2_O with H abstracted from benzene, indicating that the benzene hydroxylation reaction proceeded in a push–pull way. This research provided a new perspective for the deep understanding of the mechanism of benzene hydroxylation to phenol over Pt-WO_3_.

In comparison with TiO_2_, ZnO is a direct band-gap semiconductor with more oxygen vacancies on its surface, which benefits the formation of electron traps and prolongs the lifetime of charge carriers, thus absorbing a wider range of the solar spectrum [52]. Sathu et al. [53] prepared inorganic leaves composed of ZnO by intercalating Zn^2+^ ions into the porous channels of magnolia tree leaves (IL-ZnO). Compared with commercial ZnO, the amounts of defects related with the IL-ZnO were effectively suppressed. In addition, the diffusion of charge carriers resulted from the preservation of nanospace and nanoarchitecture further improved the catalytic performance of IL-ZnO in benzene hydroxylation under UV-light.

Hierarchical nanostructures with specific morphology have attracted specific attention in recent years [54]. Chen et al. [55] prepared a novel three-dimensional (3D) Bi_2_WO_6_/CdWO_4_ (BCW) through the decoration of CdWO_4_ micro rods with Bi_2_WO_6_ nanosheets. Due to the unique hierarchical heterostructure, which facilitated the absorption of visible light and separation of photogenerated carriers, BCW exhibited a high phenol selectivity (>99%). The FeVO_4_ nanorods grafted with covalently bonded organosilane (OS) groups was explored by Wei et al. for the photocatalytic hydroxylation of benzene [56]. The OS groups grafted on FeVO_4_ could not only modify the surface affinity of FeVO_4_ to enhance the benzene adsorption and phenol desorption ability, but also act as an effective protective coating to suppress metal leaching with maintaining the visible light response ability of FeVO_4_, therefore, resulting in an excellent photocatalytic performance in the benzene to phenol reaction.

The results of photocatalytic hydroxylation of benzene to phenol using different types of semiconductors are summarized in Table 1.

### 2.2. POMs-Based Photocatalysts

POMs are anionic nanoclusters of transition metal oxides with a variety of structures [57,58]. Due to their high oxidation stability, excellent water solubility, and unique structure-dependent reversible redox properties, POMs have been exploited as a versatile class of redox reagents for photocatalytic hydroxylation of benzene to phenol (summarized in Table 2). In order to enhance the stability and reusability of homogeneous POMs catalytic systems, POMs-based heterogeneous catalysts have been fabricated via various “immobilization” or “solidification” strategies. Zhang et al. [59] conducted benzene oxidation with functional POMs paired ionic salts (IL-POMs), which were prepared by pairing quinoline cations with Keggin-type phosphotungstic (PW) anions. It was described that the solubility of these IL-POMs was dependent on the length of the carbon chain in the alkyl groups of quinoline cations, and heterogeneous photocatalysts could be formed only with a long carbon chain. Due to the suppressed recombination of photo-induced carriers benefitting from the unique redox property of POM anions, IL-POMs showed a higher phenol yield (20.9%) than the quinoline salt precursor and phosphotungstic (PW) alone. Xu et al. [60] realized the direct oxidation of benzene to phenol with NH_2_-MIL-88/PMo_10_V_2–3_. In their research, a K-type vanadium-substituted POMs (PMo_10_V_2_) was immobilized on amine-functionalized MIL-88 (NH_2_-MIL-88) to fabricate a stable heterogeneous photocatalyst, in which heteropoly acid anions were able to “grab” NH^3+^ ions. The obtained NH_2_-MIL-88/PMo_10_V_2_ exhibited outstanding catalytic performance, which would be attributed to the high dispersion of PMo_10_V_2_, •OH radicals generation and the V^5+^/V^4+^ redox pairs formed in situ in the presence of electrons (e^−^). Recently, Gu et al. [61] reported the development of supramolecular catalysts based on vanadium-substituted POMs anion and quinolinium ions for the oxidation of benzene to phenol. A vanadium-substituted POMs anion was found to be able to not only stabilize quinolinium radicals but also reuse H_2_O_2_ produced by quinolinium ions, to offer a high phenol yield of 50.1%. However, the reusability of the as-prepared supramolecular catalysts remained a concern because of the presence of quinolinium ions.

### 2.3. g-C_3_N_4_-Based Photocatalysts

As a class of N-doped polymeric materials, carbon nitride (CN) has attracted worldwide attention owing to its prominent performance as catalyst or catalyst support [62,63]. Among various nanostructured CNs, the weakly ordered g-C_3_N_4_ is recognized as the most stable one under ambient conditions and can be prepared from low-cost nitrogen-rich precursors (e.g., melamine, dicyandiamide, urea, etc.) [64]. Furthermore, a stacked 2D structure and a suitable band gap (2.7 eV) enable g-C_3_N_4_ to be utilized in photocatalytic oxidation reactions [65]. In particular, porous g-C_3_N_4_ was found could chemically adsorb and activate benzene [66]. However, the photocatalytic activity of pristine g-C_3_N_4_ was not ideal, owing to its low surface area and the fast recombination of the photo-induced carriers. So far, non-metallic and metallic materials have been employed to dope in g-C_3_N_4_, to promote its photocatalytic performance for hydroxylation of benzene (as summarized in Table 3).

As an important modification strategy, fluorination has been utilized to modify graphite, carbon nanotubes, boron nitride nanotubes, activated carbon, etc. [79] The fluorinated polymeric carbon nitride solids (CNFs) have shown promising application potential in photo-catalysis. As reported by Wang et al. NH_4_F was directly incorporated into the thermally induced CN solids [67] to adjust the electronic band gaps and redox properties of the resultant catalyst, which resulted in an improved conversion of benzene to phenol, although the photocatalytic activity was still limited. In order to expose catalytic sites, Fe^3+^ was used to be doped on g-C_3_N_4_, followed by coated-on mesoporous Santa Barbara Amorphous-15 (SBA-15) to obtain a porous catalyst, with which a phenol yield of up to 11.9% [68] could be realized. To further investigate the catalytic mechanism of the Fe/g-C_3_N_4_ in benzene oxidation, especially the interaction between Fe and g-C_3_N_4_, Zhang et al. [69] conducted the photocatalytic hydroxylation reaction over mesoporous g-C_3_N_4_ hybrids (FeCl_3_/mpg-C_3_N_4_) under visible light illumination. By optimizing the loading amount of FeCl_3_ in the catalyst, the activity of the catalyst was effectively enhanced, which was attributed to the promoted redox cycle of Fe^2+^/Fe^3+^. With high surface area and long-range ordered pores, mesoporous-type Laval University Silica (LUS-1) has been employed as a support for loading g-C_3_N_4_, to improve its photocatalytic activity. In the prepared Fe-g-C_3_N_4_-LUS-1, a single layer of g-C_3_N_4_ was formed on the surface of LUS-1, and 16% of the phenol yield could be obtained under sunlight [70].

Fe-g-C_3_N_4_ (Fe-CN)/titanium silicate zeolite (TS-1) composites were also employed for the hydroxylation of benzene to phenol under visible light irradiation [71]. It was revealed that Fe doping could promote photocatalytic activity and give a phenol yield that was ~9 times and ~4 times higher than single Fe-CN and TS-1, respectively. The effect of metal deposited in the composites (M-CN/TS-1) was also examined for phenol production. Results showed the catalytic activities of different metal-deposited catalysts were in the following order: Fe-CN/TS-1 > Cu-CN/TS-1 > Ni-CN/TS-1 > Zn-CN/TS-1 > Co-CN/TS-1. However, due to the weak chemical interaction between the host and Fe-CN guest, both Fe-g-C_3_N_4_/SBA-15 and Fe-CN/TS-1 encountered low catalytic stability associated with the host–guest separation in the reaction. To avoid this circumstance, ferrocene carboxyaldehyde (Fc-CHO) was immobilized on the surface of mesoporous graphitic carbon nitride (MCN) via a covalent C=N linkage, to form a stable π-conjugation system [72]. It was described that the synergistic donor-acceptor interaction between the CN matrix and Fc group could not only enhance excited electrons splitting but also act as an effective electron sink, supporting iron-cascade catalysis. Therefore, the polymeric material Fc-MCN exhibited superior photocatalytic performance in the benzene oxidation reaction to unmodified mpg-C_3_N_4_ and Fc-CHO. In another work [73], a new polymeric Fc-CO-NH-C_3_N_4_ (Fc-CN) material was synthesized by the amidation of ferrocene carboxylic acid (Fc-COOH) with -NH_2_ groups on the surface of MCN, and a phenol yield of 10% was achieved. Owing to its binary structure, the polymeric Fc-CN can not only expand the absorption range of visible light but also facilitate the separation and migration of excited charge carriers to the surface of the catalyst.

**Figure 4:**
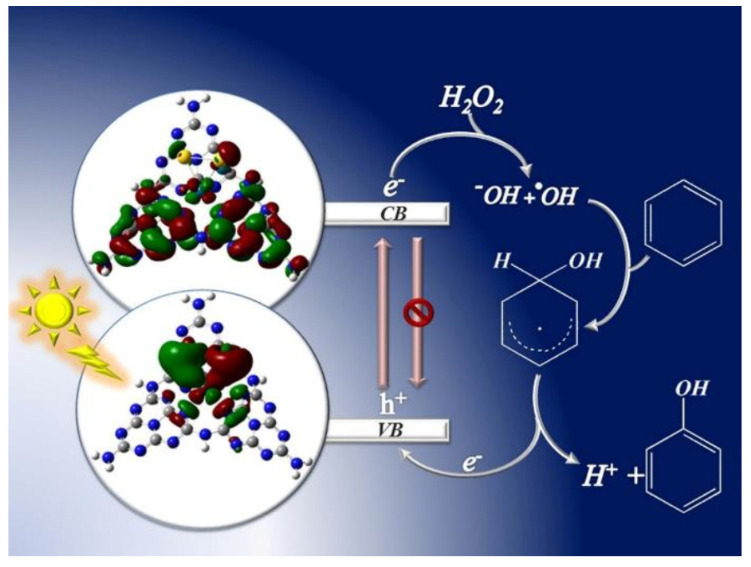
Proposed mechanism for hydroxylation of benzene by Au-Pd/g-C_3_N_4_ catalyst under visible-light irradiation. Reprinted with permission from Ref. [77]. 2018 American Chemical Society.

As demonstrated by Ding et al., the electronic, optical and catalytic properties of g-C_3_N_4_ were highly adjustable through metal doping. In their research, transition metals including Fe, Co, Ni, Mn, and Cu were incorporated into the g-C_3_N_4_ matrix via a simple soft-chemical approach [74]. Fe-g-C_3_N_4_ and Cu-g-C_3_N_4_ exhibited higher performance than Mn−, Ni−, and Co− modified g-C_3_N_4_ in the hydroxylation of benzene under mild conditions. Bimetal including Cu-Ag, Cu-Au, and Au-Pd were also employed to immobilize on g-C_3_N_4_ for the photocatalytic hydroxylation of benzene to phenol, and the reaction temperature could be significantly brought down. As for CuAg@g-C_3_N_4_, the synergistic effect between Cu and Ag was thought to play a vital role in the activation of benzene and production of active •OH radicals [75]. In addition, loading Cu and Au nanoparticles (NPs) on g-C_3_N_4_ with a large surface area could further improve the overall dispersion of metal NPs, thereby enhancing the catalytic performance of the bimetallic catalysts. Under this condition, benzene was completely converted to phenol (up to 99% conversion), without the formation of any by-products [76]. While for Au-Pd@g-C_3_N_4_, the Au-Pd nanoparticles were incorporated into the g-C_3_N_4_, electrons transferred from the HOMO of Au-Pd nanoparticles to the LUMO of g-C_3_N_4_, which prolonged the lifetime of the excitons and reduced the charge-hole recombination, thereby increasing phenol yield and selectivity over Au-Pd@ g-C_3_N_4_ (Figure 4) [77].

In a recent work by Basyach et al., g-C_3_N_4_ was blended with Ni-doped CuWO_4_ nanoparticles to prepare a Z-scheme Ni-CuWO_4_/g-C_3_N_4_ nanocomposite. Due to the narrow band gap between the Ni-CuWO_4_/g-C_3_N_4_ nanocomposite and the enhanced visible light absorption in a specific wavelength range, the recombination of photogenerated holes and electrons was minimized, and, therefore, higher benzene conversion and phenol yield could be obtained than with pristine Ni-CuWO_4_ under sunlight [78]. Although the g-C_3_N_4_-based photocatalysts described above exhibited enhanced performance in hydroxylation of benzene, their application was restricted, since urea was usually employed as the precursor for g-C_3_N_4_ preparation, which suffered from extremely low productivity (<10 wt%).

### 2.4. MOFs-Based Photocatalysts

MOFs are a broad family of crystalline micro-mesoporous hybrid materials that have emerged as fascinating photocatalysts owing to their large surface areas, excellent stability, and uniform-but-tunable cavities [80]. The light irradiations on MOFs will lead to the generation of electrons and holes that can participate in the redox reactions, even though their mobilities are relatively lower compared with those of semiconductors [81]. In 2015, Wang et al. reported the first exploitation of MOFs for photocatalytic benzene hydroxylation, in which two water-stable Fe-based MOFs, MIL-100(Fe) and MIL-68(Fe), were prepared [82]. It could be concluded from electron spin resonance (ESR) and kinetic studies that the photocatalysis of the Fe-O clusters in Fe-based MOFs, combined with H_2_O_2_ oxidized to radicals (•OH) via a Fenton-like route, was involved in the process. Moreover, MIL-100(Fe) exhibited superior photocatalytic performance, indicating that the microstructure of the MOFs could significantly affect the photocatalytic efficiency. Inspired by this work, Xu et al. [83] prepared nanoscale MIL-100(Fe) particles using ethylene glycol for the first time. Owing to their porosity structure and high surface area, MIL-100(Fe) nanoparticles provided higher H_2_O_2_ efficiency (58.5%) than MIL-68(Fe). In a later work, Cu^II^-based MOF was prepared for photocatalytic hydroxylation of benzene, and a Fenton oxidation mechanism was proposed [84]. In their proposed mechanism, an excited charge separation occurred in the Cu^II^-based MOFs under visible light, affording an electron for the reduction of Cu^2+^, which was then reduced to Cu^+^. The newly formed Cu^+^ can reduce H_2_O_2_ to •OH radicals under acidic conditions, while Cu^+^ was oxidized back to Cu^2+^. According to ESR studies, the generated •OH radicals were necessary during the catalytic process.

In recent years, various post-synthetic chemical treatments have been employed for the organic functionalization of MOFs. Fang et al. [85] prepared a novel heterogeneous catalyst UiO-66-NH_2_-SA-V, by anchoring VO(acac)_2_ on the Schiff base UiO-66-NH_2_-SA via chemical bonds. With acetonitrile and acetic acid used as solvent, a phenol selectivity of 100% and a phenol yield of 15.3% could be achieved. The excellent catalytic performance of UiO-66-NH_2_-SA-V was attributed to the vanadium species, with high catalytic activity, and the interaction between the Zr-MOF support and benzene molecules. In a later work [86], the UiO-66-NH_2_ was replaced with NH_2_-MIL-88B(Fe) to prepare a high-performance catalyst NH_2_-MIL-88B(Fe)-SA-V, which could effectively promote the adsorption of benzene on the catalyst to generate phenol and further improve the yield of phenol to 22.2%.

The catalytic performance of various MOFs-based catalysts for benzene hydroxylation are summarized in Table 4.

### 2.5. Carbon Materials-Based Photocatalysts

Carbon materials have shown wide applications in the field of organic reactions as support and catalytic active materials because of their unique properties and high stability [11,87]. As mentioned earlier, the increased reactivity of phenol compared to benzene leads to the further oxidation of the phenol, which will lower its selectivity. In this regard, the high adsorption capacity and interaction affinity toward benzene are the key factors for efficient photocatalysts. Furthermore, as the hydroxylation of benzene is a typical conversion of a hydrophobic reactant to a hydrophilic product, a promising strategy to enhance the benzene-adsorption capability of the catalyst is to improve its surface hydrophobicity. The aforementioned catalysts are generally hydrophilic and have a weak interaction affinity with benzene, which hinders the benzene’s activation. In this context, several types of carbon materials, including multi-walled carbon nanotubes (CNT), activated carbon, graphene, reduced graphene oxide (RGO), etc., were developed to increase the hydrophobicity of the catalyst and avoid further degradation of phenol.

**Figure 5:**
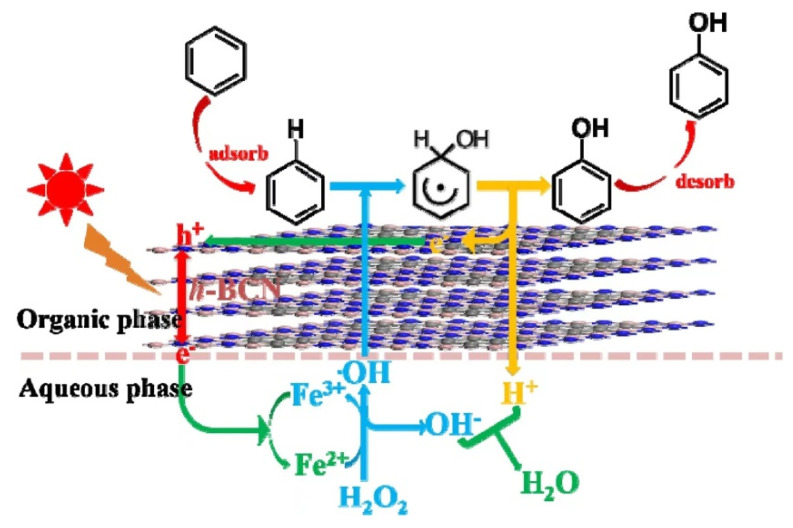
Reaction mechanism of photocatalytic hydroxylation of benzene by h-BCN combined with FeCl_3_. Reprinted with permission from Ref. [88]. 2019 Elsevier.

Wang et al. [88] prepared ternary hexagonal boron carbon nitride (h-BCN) nanosheets by in situ doping of biomass glucose into hexagonal boron nitride (h-BN). The ternary 2D h-BCN nanosheets, which combined the advantages of graphene and h-BN, were found to possess tunable energy band and exhibit a unique adsorption property toward benzene. In the reaction system, benzene in the CH_3_CN phase was strongly adsorbed on the surface of h-BCN, while FeCl_3_ and H_2_O_2_ were both in the aqueous phase. In such a reaction situation, h-BCN existed at the interface between the organic phase and aqueous phase, which kept the benzene molecules well adsorbed on h-BCN and effectively reacted with the •OH generated by the photo-Fenton reaction, to increase the conversion of benzene to phenol. The weaker adsorption ability of phenol on h-BCN make the phenol easily desorbed from the surface of h-BCN and then solubilized into the organic phase, thus preventing the over-oxidation of the phenol (Figure 5).

Among all the reported carbon-based materials, graphene and RGO materials have been extensively reported to have notable activity in photocatalysis [89,90]. Cai et al. [91] have demonstrated that through the conversion of the surface wettability of RGO from hydrophilic to hydrophobic (RGO-Cys), the benzene hydroxylation reaction occurred at the water–benzene interface, and the rapid desorption of phenol from the interface to the benzene phase was realized, thereby significantly enhancing the photocatalytic performance by more than three times. In a study by He et al., surface-modified Cu_2_O supported on defective graphene was prepared for the selective photocatalytic hydroxylation of benzene [92]. The surface hydrophobicity of the catalyst was increased through the modification of alkanethiols to promote the adsorption of the benzene, which largely improved the selectivity of phenol compared to that of the catalyst without surface modification.

**Figure 6:**
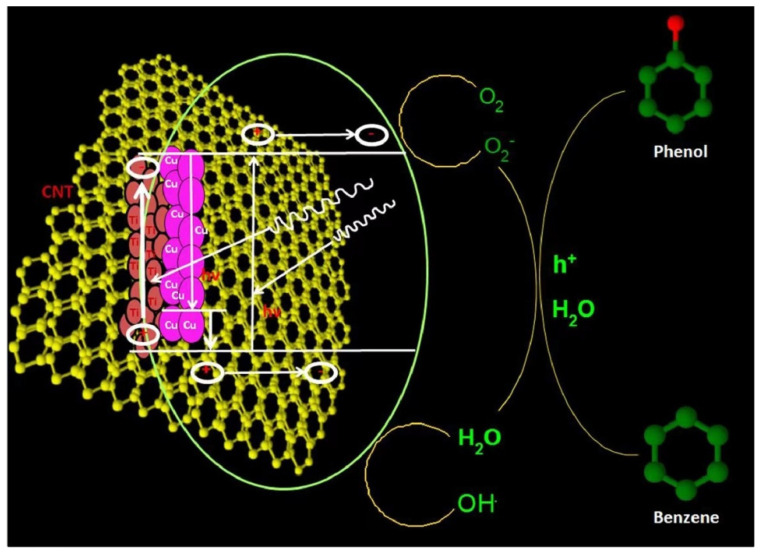
Proposed mechanism of photocatalytic benzene oxidation to phenol over copper/titanium dioxide/CNT catalysts. Reprinted with permission from Ref. [93]. 2018 Elsevier.

An enhanced phenol selectivity was obtained by supporting Cu impregnated TiO_2_ with CNT [93]. The strong interphase interaction between Cu, TiO_2_, and CNTs could not only extend the light absorption of composites to longer wavelengths but also lead to an improved benzene-adsorption capacity and, therefore, enhance the sequential interaction between the hydroxyl radicals and the adsorbed benzene on copper/titania surfaces. As depicted in Figure 6, the mechanism of photocatalytic benzene oxidation over Cu/TiO_2_/CNTs was proposed, in which the following processes were involved: the photo excited electrons of CNTs transferred into the CB of TiO_2_ enabling the production of highly reactive peroxide radicals, and positively charged CNT might grab electrons from the VB of TiO_2_, with holes reserved for the formation of •OH radicals from H_2_O.

**Figure 7:**
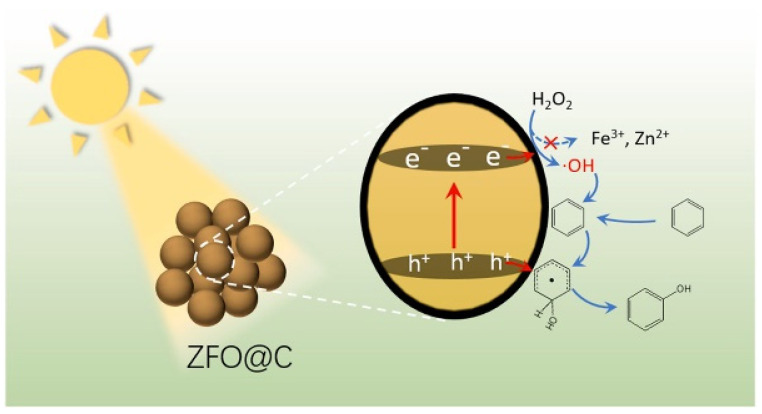
Photocatalytic hydroxylation of benzene to phenol over ZFO@C. Reprinted with permission from Ref. [95]. 2022 Elsevier.

Spinel ZnFe_2_O_4_ (ZFO) has also shown catalytic behavior toward H_2_O_2_ activation via a photo-Fenton route [94]. However, the rapid charge recombination, inevitable metal leaching, and hydrophilic surface structure greatly limited its catalytic performance. The encapsulation of spinel ZFO@C by ultrathin carbon was conducted by Yang and coworkers [95], which not only protected the ZFO@C from corrosion and metal leaching but also increased the surface affinity for benzene molecules. The hydrophobic carbon with a π-conjugated electron system was beneficial for the adsorption of benzene instead of phenol, which consequently facilitated the conversion of benzene to phenol (Figure 7). 

Recently, the use of N-doped carbon layer to encapsulate iron nanoparticles (Fe@NC) has been adopted to functionalize the iron-containing catalyst for photocatalytic benzene hydroxylation, which benefited from its unique core-shell nanostructure as well as strong host–guest electronic interactions between iron and carbon [96]. Due to the excellent stability against acid etching and the hydrophobic surface properties of rigid carbon shells, the synthesized Fe@NC promoted the adsorption of benzene and exhibited excellent catalytic durability and high selectivity.

The catalytic performance of various carbon-materials-based photocatalysts for hydroxylation of benzene are summarized in Table 5.

### 2.6. Other Photocatalysts

Besides the aforementioned heterogeneous photocatalysts, other types of materials have also been developed to improve the catalytic performance of benzene hydroxytion [97,98,99] (as summarized in Table 6). Layered double hydroxide (LDH), as a class of 2D inorganic layered matrix, was incorporated with specific photoactive Zn^2+^/Ti^4+^ to prepare Zn_2_Ti-layered double hydroxide (ZnTi-LDH) [98], and a phenol selectivity of 87.18% was achieved. By employing the strategies of band structure tailoring and defect engineering, the VB position of ZnTi-LDH was appropriate to match the oxidation potential of benzene, and the sufficient oxygen vacancies (VO) were beneficial for improving electron-hole separation efficiency as well as the formation of superoxide radical anion (O^2−^), thus resulting in excellent catalytic performance. As transition metal complexes, especially iron complexes, have been regarded as the high-performance catalysts for various oxidation reactions, a cyano-bridged polynuclear metal complex containing Fe(II) and Ru(II) incorporated in SAl-MCM-41 ([Fe(H_2_O)_3_]_2_[Ru(CN)_6_]@sAl-MCM-41) [99] and Fe(II) phthalocyanine [97] were reported as the heterogeneous photocatalysts for the selective oxidation of benzene to phenol, in which the catalytic performance was rival to that of noble metal catalysts.

## 3. Conclusions

Photocatalytic oxidation of benzene has shown a promising future, with several distinct advantages for phenol synthesis. It is well-documented that the efficacy of a photocatalyst is greatly dependent on its intrinsic characteristics, which can be selectively adjusted through appropriate modifications, and a slight change of physicochemical properties could arouse a significant decrease in photocatalytic activity. Thereby, the design and fabrication of a highly selective and efficient photocatalyst is crucial for selective benzene oxidation. This review documented the tremendous progress that has been achieved in the development of various heterogeneous photocatalysts, including semiconductors, POMs, g-C_3_N_4_, MOFs, carbon materials, etc., which exhibited significant benzene conversion and phenol selectivity. However, some challenges related to photocatalysts remain to be addressed. First, the performances of heterogeneous photocatalysts were usually restricted by a lower catalytic activity associated with their homogeneous counterparts. Additionally, some of the applied synthetic approaches are very complicated and difficult to be scalable. Besides, the unavoidable leaching of the active components in the metal doped heterogeneous photocatalysts should also be taken into consideration. It is, thus, highly desirable to explore simple, stable, efficient, and, particularly, cost-effective heterogeneous catalysts for industrial applications. In this regard, it is imperative to explore novel and facile methods for catalyst modification that aim to improve the benzene-adsorption capacity and photocatalytic activity. Finally, new insights into the catalytic mechanism will also be necessary, and it will provide inspirations to the design and fabrication of more distinctive and excellent visible-light-responsive photocatalysts for benzene hydroxylation, which is expected to have many potential applications in industrial phenol production.

## Figures and Tables

**Table 1:** Catalytic performance of semiconductor-based photocatalysts for benzene hydroxylation.

Photocatalyst	Reaction Conditions	Con./%	Sel./%	Yield/%	Ref.
[Ru(bpy)_3_]^2+^–SSA@TiO_2_ (0.42 g/L)	Simulated solar, benzene (600 ppm), phenol (83,000 ppm), H_2_O, 5 h	72	96	63.5	[31]
mTiO_2_ (10 mg)	λ > 320 nm, 0.02 mmol benzene, 10 mL H_2_O, 40 °C, 6 h	42	81	34	[32]
H-MAG TiO_2_ (120 mg)	Simulated solar λ > 320 nm, aqueous benzene (20 mL H_2_O, saturated with O_2_), 42 °C, 24 h	80	100	80	[33]
Na-MAG TiO_2_ (120 mg)		80	28	22.7	
TiO_2_@MCF (1 g/L)	λ > 320 nm, 29.7 mL H_2_O, 0.3 mL acetonitrile, 10 mmol benzene, RT, 2 h	72	30.7	22.2	[34]
Au@TiO_2_ (50 mg)	Xe arc lamp λ > 400 nm, 0.07 mL benzene, 50 mL H_2_O, 30 °C, 3 h	65	96	62	[35]
Au/TiO_2_ (60 mg)	λ > 320 nm, 60 ppm benzene, 230 Kpa CO_2_, 20 mL H_2_O, 34 °C, 24 h	15	89	13	[36]
Au/TiO_2_ (60 mg)	Hg lamp (240–440 nm), 5 mL benzene, 500 mL H_2_O, 25 °C, 6 h				[37]
Au @TiO_2_ (2 g/L)	λ > 400 nm, 50 mL H_2_O, 0.07 mL benzene, RT, 2 h	69	91	63	[38]
Pt @TiO_2_ (50 mg)		60	52	31	
Ag@TiO_2_ (50 mg)		0.3	0.9	0.3	
Au_shell_-Pd_core_/TiO_2_ (5 mg/L)	UV LED light, 1 mmol/L benzene and H_2_O (100 mL), RT, 1 h	30			[39]
Au/Ti_0.98_V_0.02_O_2_ (30 mg)	Hg lamp (200–400 nm), 2 mL benzene, 1 mL H_2_O_2_, 1 mL CH_3_CN, 25 °C, 18 h	18	88.1	15.9	[40]
Pd/CeO_2_/TiO_2_ (100 mg)	Xe lamp λ > 420 nm, 1 g benzene, 10 mL CH_3_CN, benzene:H_2_O_2_ (molar ratio) = 1:5, 80 °C, 10 h	73	95	69.4	[41]
TiO_2_+POM (25 mg)	Xe arc lamp λ > 300 nm, benzene:H_2_O:CH_3_CN = 0.05 mL:24 mL:1 mL, O_2_, RT, 8 h	13	85	11.0	[42]
Ti_0.98_Fe_0.01_Cr_0.01_O_2_ (30 mg)	Hg lamp (200–400 nm), 1 mL benzene,2 mL CH_3_CN, 2 mL H_2_O_2_, H_2_O, 25 °C, 3–18 h	15	94	14.1	[43]
FeVCu/TiO_2_ (10 mg)	Black light blue fluorescent bulb, benzene:H_2_O_2_ = 0.5, 40 mL CH_3_CN, 30 °C, 4 h	18.6	52	9.7	[44]
Cu(OH)_2_/LT (5 mg)	UV illumination, 100 μL benzene, 500 μL CH_3_CN, 13 mL H_2_O, 87 μL H_2_O_2_, RT, 6 h	50	97	45	[45]
Pt/WO_3_ (20 mg)	220 < λ < 470 nm, 1 mL benzene, 1 mL H_2_O, 60 °C, 3 h		97		[48]
Pt(0.2)-WO_3_ (20 mg)	300 < λ < 500 nm, 2.5 mmol benzene, 7.5 mL H_2_O, 60 °C, 4 h	69	74	49	[49]
Pt/WO_3_ (20 mg)	Xe lamp (420–540 nm), 0.3 mmol benzene, 5 mL H_2_O, 25 °C, 20 h		70		[51]
IL-ZnO_2_ (25 mg)	Hg lamp (250–450 nm), 1 mL benzene, 2 ml CH_3_CN, 2 mL H_2_O_2_, 25 °C, 12 h	5.2	92	4.8	[53]
Bi_2_WO_6_/CdWO_4_ (50 mg)	Xe lamp (λ > 420 nm), 3 mL CH_3_CN, 0.1 mL H_2_O, 0.5 mmol benzene, 25 °C, 3 h	7.3	99	7.2	[55]
FeVO_4_@TMOS (30 mg)	Xe lamp (λ > 420 nm), 3 mL CH_3_CN, 3 mL H_2_O, 0.1 mL benzene, 2 mL H_2_O_2_, 24 °C, 4 h	20	98	20	[56]
FeVO_4_@DTOS (30 mg)		13	98	13	

**Table 2:** Catalytic performance of POMs-based photocatalysts for benzene hydroxylation.

Photocatalyst	Reaction Conditions	Con./%	Sel./%	Yield/%	Ref.
IL-POMS (25 μmol)	Xe lamp (λ > 420 nm), 1.28 mmol benzene, 10 mL CH_3_CN, 1 mL H_2_O, RT, 10 h.	21	99	20.9	[59]
NH_2_-MIL-88/ PMo_10_V_2_ (20 mg)	LED lamp (320–780 nm), 1 mL benzene, 3 mL acetic acid, 3 mL CH_3_CN, 1 mL H_2_O_2_, 60 °C, 3 h	12.5	99	12.4	[60]
Quinolinium and Polyoxovanadate-Based Supramolecular (0.0125 mmol)	Xe lamp (λ > 420 nm), H_2_O:CH_3_CN = 3:17(*v*:*v*), 0.5 mmol benzene, 25 °C, 12 h	51	99	50.8	[61]

**Table 3:** Catalytic performance of g-C_3_N_4_-based photocatalysts for benzene hydroxylation.

Photocatalyst	Reaction Conditions	Con./%	Sel./%	Yield/%	Ref.
CNF (50 mg)	Xenon lamp (λ > 420 nm), 0.8 mL benzene, 4 mL H_2_O, 4 mL CH_3_CN, 0.51 mL H_2_O_2_, 60 °C, 4 h		16.8		[67]
Fe-g-C_3_N_4_/SBA-15 (50 mg)	Xenon lamp (λ > 420 nm), 0.8 mL benzene, 4 mL H_2_O, 4 mL CH_3_CN, 0.51 mL H_2_O_2_, 60 °C, 4 h			11.9	[68]
FeCl_3_/mpg-C_3_N_4_ (25 mg)	Mercury lamp, 4.5 mmol benzene, 2 mL H_2_O, 2 mL CH_3_CN, 0.255 mL H_2_O_2_, 60 °C	38	97	32.5	[69]
Fe-g-C_3_N_4_-LUS-1 (50 mg)	Mercury lamp, 4 mL CH_3_CN, 1 mL benzene, 0.5 mL H_2_O_2_, 4 h, 60 °C		98		[70]
Fe-CN/TS-1 (50 mg)	Xenon lamp (λ > 420 nm), 0.8 mL benzene, 4 mL H_2_O, 4 mL CH_3_CN, 0.5 mL H_2_O_2_, 60 °C, 4 h	54	18.4	10	[71]
Fc-MCN (50 mg)	Xenon lamp (λ > 420 nm), 0.8 mL benzene, 4 mL H_2_O, 4 mL CH_3_CN, 0.51 mL H_2_O_2_, 60 °C, 4 h	48	34.7	16.5	[72]
Fc-CN (50 mg)	Xenon lamp (λ > 420 nm), 0.8 mL benzene, 4 mL H_2_O, 4 mL CH_3_CN, 0.51 mL H_2_O_2_, 60 °C, 4 h			16.9	[73]
Fe-g-C_3_N_4_ (50 mg)	Xenon lamp (λ > 420 nm), 0.8 mL benzene, 4 mL H_2_O, 4 mL CH_3_CN, 0.51 mL H_2_O_2_, 60 °C, 4 h	100	8.3	8.3	[74]
Cu-g-C_3_N_4_ (50 mg)		76.7	3.6	2.6	
Ni-g-C_3_N_4_ (50 mg)		12	1.7	0.2	
Mn-g-C_3_N_4_ (50 mg)		42.9	0.15	6.2	
Co-g-C_3_N_4_ (50 mg)		40.2	0.003	0.002	
Cu-Ag@g-C_3_N_4_ (25 mg)	Domestic bulb, 1 mmol benzene, 5 mL CH_3_CN, 1.1 mmol H_2_O_2_, RT, 30 min	99			[75]
Cu-Au@g-C_3_N_4_ (50 mg)	Cool LED bulb, 1 mmol benzene, 5 mL CH_3_CN, 1.1 mmol H_2_O_2_, RT, 30 min	99			[76]
Au-Pd@g-C_3_N_4_ (10 mg)	Mercury lamp (λ > 420 nm), 1 mL benzene,5 mL CH_3_CN, 2 mL H_2_O_2_, 50 °C, 2 h	26	100	26	[77]
Ni-CuWO_4_/g-C_3_N_4_ (20 mg)	Sunlight, 1 mL benzene, 0.2 mL H_2_O, 0.5 mL H_2_O_2_, 15 min	98.5	81.5	80.3	[78]

**Table 4:** Catalytic performance of MOFs-based photocatalysts for benzene hydroxylation.

Photocatalyst	Reaction Conditions	Con./%	Sel./%	Yield/%	Ref.
MIL-100(Fe) (10 mg)	Xenon lamp (λ > 420 nm), 0.5 mmol benzene, 2 mL H_2_O, 2 mL CH_3_CN, 0.375 mmol H_2_O_2_, RT, 8 h	20.1	98	19.5	[82]
MIL-100(Fe) (25 mg)	Xenon lamp (λ > 420 nm), 1 mmol benzene, 5 mL H_2_O, 3 mL CH_3_CN, 0.6 mmol H_2_O_2_, RT, 3–21 h	34.4	98	33.8	[83]
Cu (II) MOF (10 mg)	LED lamp (λ > 420 nm), 1 mmol benzene, 10 mL H_2_O, 1.25 mmol H_2_O_2_, 60 °C, 10 h	29	95	27.4	[84]
UiO-66-NH_2_-SA-V (10 mg)	Xenon lamp (λ > 420 nm), 1 mL benzene, 5 mL CH_3_CN, 1 mL acetic acid, H_2_O_2_, 60 °C, 4 h	15.3	100	15.3	[85]
NH_2_-MIL-88B(Fe)-SA-V (30 mg)	Visible light, 1 mL benzene, 18 mL acetic acid, H_2_O_2_, 60 °C, 4 h	22.5	98.6	22.2	[86]

**Table 5:** Catalytic performance of carbon-materials-based photocatalysts for benzene hydroxylation.

Photocatalyst	Reaction Conditions	Con./%	Sel./%	Yield/%	Ref.
h-BCN (50 mg)	Xenon lamp (λ > 420 nm), 0.8 mL benzene, 4 mL FeCl_3_ (aq), 4 mL CH_3_CN, 0.5 mL H_2_O_2_, 60 °C, 2 h	16	88.3	14	[88]
RGO-Cys (60 mg)	LED lamp, 5 mmol benzene, 25 mL H_2_O, 5 mmol H_2_O_2_, 60 °C, 20 h	1.0	87	0.87	[91]
Cu_2_O/dG (5 mg)	ED lamp, 1 mmol benzene, 5 mL H_2_O, 1 mmol H_2_O_2_, 25 °C, 16 h	30	63.9	19.3	[92]
Cu/Ti/CNT (100 mg)	Mercury lamp UV–vis, 20 mL benzene, 20 mL H_2_O, 1 mmol H_2_O_2_, 70 °C	68.3	75.8	51.8	[93]
ZFO@C (30 mg)	Xenon lamp (λ > 420 nm), 0.1 mL benzene, 3 mL CH_3_CN, 3 mL H_2_O, 0.5 mL H_2_O_2_, RT	16	99.4	15.5	[95]
Fe@NC (30 mg)	0.25 mL benzene, 3 mL H_2_O, 3 mL CH_3_CN, 2 mL H_2_O_2_, 60 °C, 12 h	16	95	14.5	[96]

**Table 6:** Catalytic performance of other photocatalysts for benzene hydroxylation.

Photocatalyst	Reaction Conditions	Con./%	Sel./%	Yield/%	Ref.
FePc (30 mg)	mercury lamp, 1 mL benzene, 3 mL H_2_O_2_, 5 mL CH_3_CN, RT, 6 h	15	99	15.2	[97]
ZnTi-LDH (20 mg)	Xe lamp (λ > 420) nm, 0.2 mmol benzene, 1 atm air, 20 mL H_2_O, 3 h, 48 °C	5.7	87.18	5.0	[98]
[Fe(H_2_O)_3_]_2_[Ru(CN)]_6_ (5 mg)	Λ > 390 nm, 2.5 mL CH_3_CN, 0.40 mL benzene, 0.40 mL H_2_O_2_, 50 °C	61.28			[99]

## References

[1] Solyman W., Nagiub H.M., Alian N.A., Shaker N.O., Kandil U.F. (2017). Synthesis and characterization of phenol/formaldehyde nanocomposites: Studying the effect of incorporating reactive rubber nanoparticles or Cloisite-30B nanoclay on the mechanical properties, morphology and thermal stability. J. Radiat. Res. Appl. Sci..

[2] Takeichi T., Furukawa N., Moeller M., Matyjaszewski K. (2012). Epoxy Resins and Phenol-Formaldehyde Resins. Polymer Science: A Comprehensive Reference.

[3] Pryde C.A., Hellman M.Y. (1980). Solid state hydrolysis of bisphenol-A polycarbonate. I. Effect of phenolic end groups. J. Appl. Polym. Sci..

[4] Schmidt R.J. (2005). Industrial catalytic processes—Phenol production. Appl. Catal. A.

[5] Zhuo Y., Zhong Y., Xu Y., Sha Y. (2016). Evaluation of Transfer Resistances in the Reactive Distillation Process for Phenol Production. Ind. Eng. Chem. Res..

[6] Jiang T., Wang W., Han B. (2013). Catalytic hydroxylation of benzene to phenol with hydrogen peroxide using catalysts based on molecular sieves. New J. Chem..

[7] Fujihira M., Satoh Y., Osa T. (1981). Heterogeneous Photocatalytic Oxidation of Aromatic Compounds on TiO_2_. Nature.

[8] Zhang T., Zhang D., Han X., Dong T., Guo X., Song C., Si R., Liu W., Liu Y., Zhao Z. (2018). Preassembly strategy to single Cu-N_3_ sites inlaid porous hollow carbonitride spheres for selective oxidation of benzene to phenol. J. Am. Chem. Soc..

[9] Long Z., Chen G., Liu S., Huang F., Sun L., Qin Z., Wang Q., Zhou Y., Wang J. (2018). Synergistic combination of graphitic C_3_N_4_ and polyoxometalate-based phase-transfer catalyst for highly efficient reductant-free aerobic hydroxylation of benzene. Chem. Eng. J..

[10] Navarro R., Lopez-Pedrajas S., Luna D., Marinas J., Bautista F. (2014). Direct hydroxylation of benzene to phenol by nitrous oxide on amorphous aluminium-iron binary phosphates. Appl. Catal. A.

[11] Wen G., Wu S., Li B., Dai C., Su D.S. (2015). Active sites and mechanisms for direct oxidation of benzene to phenol over carbon catalysts. Angew. Chem. Int. Ed..

[12] Al-Sabagh A., Yehia F., Eshaq G., ElMetwally A. (2017). Eclectic hydroxylation of benzene to phenol using ferrites of Fe and Zn as durable and magnetically retrievable catalysts. ACS Sustain. Chem. Eng..

[13] Herrerías C.I., Yao X., Li Z., Li C.-J. (2007). Reactions of C-H Bonds in Water. Chem. Rev..

[14] Piera J., Baeckvall J.E. (2008). Catalytic oxidation of organic substrates by molecular oxygen and hydrogen peroxide by multistep electron transfer—A biomimetic approach. Angew. Chem. Int. Ed..

[15] Niwa S.I., Eswaramoorthy M., Nair J., Raj A., Itoh N., Shoji H., Namba T., Mizukami F. (2002). A One-Step Conversion of Benzene to Phenol with a Palladium Membrane. Science.

[16] Lee B., Naito H., Hibino T. (2012). Electrochemical Oxidation of Benzene to Phenol. Angew. Chem. Int. Ed..

[17] Ascenzi D., Franceschi P., Guella G., Tosi P. (2006). Phenol Production in Benzene/Air Plasmas at Atmospheric Pressure. Role of Radical and Ionic Routes. J. Phys. Chem. A.

[18] Shoji O., Kunimatsu T., Kawakami N., Watanabe Y. (2013). Highly selective hydroxylation of benzene to phenol by wild-type cytochrome P450BM3 assisted by decoy molecules. Angew. Chem. Int. Ed..

[19] Mukarakate C., Mcbrayer J.D., Evans T.J., Budhi S., Robichaud D.J., Iisa K., Dam J.T., Watson M.J., Baldwin R.M., Nimlos M.R. (2015). Catalytic fast pyrolysis of biomass: The reactions of water and aromatic intermediates produces phenols. Green Chem..

[20] Elkasabi Y., Mullen C.A., Boateng A.A. (2015). Aqueous extractive upgrading of bio-oils created by tail-gas reactive pyrolysis to produce pure hydrocarbons and phenols. ACS Sustain. Chem. Eng..

[21] Borah P., Ma X., Nguyen K.T., Zhao Y. (2012). A vanadyl complex grafted to periodic mesoporous organosilica: A green catalyst for selective hydroxylation of benzene to phenol. Angew. Chem..

[22] Chen L., Tang J., Song L.-N., Chen P., He J., Au C.-T., Yin S.-F. (2019). Heterogeneous photocatalysis for selective oxidation of alcohols and hydrocarbons. Appl. Catal. B.

[23] Hao H., Zhang L., Wang W., Zeng S. (2018). Modification of heterogeneous photocatalysts for selective organic synthesis. Catal. Sci. Technol..

[24] Fukuzumi S., Ohkubo K. (2015). One-Step Selective Hydroxylation of Benzene to Phenol. Asian J. Org. Chem..

[25] Ohkubo K., Kobayashi T., Fukuzumi S. (2011). Direct oxygenation of benzene to phenol using quinolinium ions as homogeneous photocatalysts. Angew. Chem. Int. Ed..

[26] Fukuzumi S., Ohkubo K. (2013). Selective photocatalytic reactions with organic photocatalysts. Chem. Sci..

[27] Ohkubo K., Fujimoto A., Fukuzumi S. (2013). Visible-Light-Induced Oxygenation of Benzene by the Triplet Excited State of 2,3-Dichloro-5,6-dicyano-p-benzoquinone. J. Am. Chem. Soc..

[28] Ohkubo K., Hirose K., Fukuzumi S. (2015). Solvent-Free One-Step Photochemical Hydroxylation of Benzene Derivatives by the Singlet Excited State of 2, 3-Dichloro-5,6-dicyano-p-benzoquinone Acting as a Super Oxidant. Chem. Eur. J..

[29] Kudo A., Miseki Y. (2009). Heterogeneous photocatalyst materials for water splitting. Chem. Soc. Rev..

[30] Fujishima A., Rao T.N., Tryk D.A. (2000). Titanium dioxide photocatalysis. J. Photochem. Photobiol. C.

[31] Goto T., Ogawa M. (2016). Efficient photocatalytic oxidation of benzene to phenol by metal complex-clay/TiO_2_ hybrid photocatalyst. RSC Adv..

[32] Shiraishi Y., Saito N., Hirai T. (2005). Adsorption-Driven Photocatalytic Activity of Mesoporous Titanium Dioxide. J. Am. Chem. Soc..

[33] Ide Y., Torii M., Sano T. (2013). Layered silicate as an excellent partner of a TiO_2_ photocatalyst for efficient and selective green fine-chemical synthesis. J. Am. Chem. Soc..

[34] Zhang G., Yi J., Shim J., Lee J., Choi W. (2011). Photocatalytic hydroxylation of benzene to phenol over titanium oxide entrapped into hydrophobically modified siliceous foam. Appl. Catal. B.

[35] Ide Y., Matsuoka M., Ogawa M. (2010). Efficient visible-light-induced photocatalytic activity on gold-nanoparticle-supported layered titanate. J. Am. Chem. Soc..

[36] Ide Y., Nakamura N., Hattori H., Ogino R., Ogawa M., Sadakane M., Sano T. (2011). Sunlight-induced efficient and selective photocatalytic benzene oxidation on TiO_2_-supported gold nanoparticles under CO_2_ atmosphere. Chem. Commun..

[37] Marino T., Molinari R., García H. (2013). Selectivity of gold nanoparticles on the photocatalytic activity of TiO_2_ for the hydroxylation of benzene by water. Catal. Today.

[38] Zheng Z., Huang B., Qin X., Zhang X., Dai Y., Whangbo M.-H. (2011). Facile in situ synthesis of visible-light plasmonic photocatalysts M@TiO_2_ (M= Au, Pt, Ag) and evaluation of their photocatalytic oxidation of benzene to phenol. J. Mater. Chem..

[39] Su R., Kesavan L., Jensen M.M., Tiruvalam R., He Q., Dimitratos N., Wendt S., Glasius M., Kiely C.J., Hutchings G.J. (2014). Selective photocatalytic oxidation of benzene for the synthesis of phenol using engineered Au–Pd alloy nanoparticles supported on titanium dioxide. Chem. Commun..

[40] Devaraji P., Sathu N.K., Gopinath C.S. (2014). Ambient Oxidation of Benzene to Phenol by Photocatalysis on Au/Ti_0.98_ V_0.02_ O_2_: Role of Holes. ACS Catal..

[41] Ma X., Dang R., Liu Z., Yang F., Li H., Guo T., Luo J. (2020). Facile synthesis of heterogeneous recyclable Pd/CeO_2_/TiO_2_ nanostructured catalyst for the one pot hydroxylation of benzene to phenol. Chem. Eng. Sci..

[42] Park H., Choi W. (2005). Photocatalytic conversion of benzene to phenol using modified TiO_2_ and polyoxometalates. Catal. Today.

[43] Devaraji P., Jo W.-K. (2018). Noble metal free Fe and Cr dual-doped nanocrystalline titania (Ti_1-x-y_M_x+y_O_2_) for high selective photocatalytic conversion of benzene to phenol at ambient temperature. Appl. Catal. A.

[44] Tanarungsun G., Kiatkittipong W., Assabumrungrat S., Yamada H., Tagawa T., Praserthdam P. (2007). Multi transition metal catalysts supported on TiO_2_ for hydroxylation of benzene to phenol with hydrogen peroxide. J. Ind. Eng. Chem..

[45] Devaraji P., Jo W.-K. (2019). Natural leaf-assisted dual-phase two-dimensional leaf TiO_2_ and Cu(OH)_2_ co-catalyst for photocatalytic conversion of benzene to phenol. Mater. Res. Bull..

[46] Szilágyi I.M., Fórizs B., Rosseler O., Szegedi Á., Németh P., Király P., Tárkányi G., Vajna B., Varga-Josepovits K., László K. (2012). WO_3_ photocatalysts: Influence of structure and composition. J. Catal..

[47] Arai T., Horiguchi M., Yanagida M., Gunji T., Sugihara H., Sayama K. (2009). Reaction mechanism and activity of WO3-catalyzed photodegradation of organic substances promoted by a CuO cocatalyst. J. Phys. Chem. C.

[48] Yoshida H., Yuzawa H., Aoki M., Otake K., Itoh H., Hattori T. (2008). Photocatalytic hydroxylation of aromatic ring by using water as an oxidant. Chem. Commun..

[49] Tomita O., Abe R., Ohtani B. (2011). Direct synthesis of phenol from benzene over platinum-loaded tungsten (VI) oxide photocatalysts with water and molecular oxygen. Chem. Lett..

[50] Tomita O., Ohtani B., Abe R. (2014). Highly selective phenol production from benzene on a platinum-loaded tungsten oxide photocatalyst with water and molecular oxygen: Selective oxidation of water by holes for generating hydroxyl radical as the predominant source of the hydroxyl group. Catal. Sci. Technol..

[51] Kurikawa Y., Togo M., Murata M., Matsuda Y., Sakata Y., Kobayashi H., Higashimoto S. (2020). Mechanistic insights into visible light-induced direct hydroxylation of benzene to phenol with air and water over pt-modified WO_3_ photocatalyst. Catalysts.

[52] Zhang L., Zhang L., Chen Y., Zheng Y., Guo J., Wan S., Wang S., Ngaw C.K., Lin J., Wang Y. (2020). CdS/ZnO: A multipronged approach for efficient reduction of carbon dioxide under visible light irradiation. ACS Sustain. Chem. Eng..

[53] Sathu N.K., Devaraji P., Gopinath C.S. (2016). Green Leaf to Inorganic Leaf: A Case Study of ZnO. J. Nanosci. Nanotechnol..

[54] Sun M.-H., Huang S.-Z., Chen L.-H., Li Y., Yang X.-Y., Yuan Z.-Y., Su B.-L. (2016). Applications of hierarchically structured porous materials from energy storage and conversion, catalysis, photocatalysis, adsorption, separation, and sensing to biomedicine. Chem. Soc. Rev..

[55] Chen P., Chen L., Zeng Y., Ding F., Jiang X., Liu N., Au C.-T., Yin S.-F. (2018). Three-dimension hierarchical heterostructure of CdWO_4_ microrods decorated with Bi_2_WO_6_ nanoplates for high-selectivity photocatalytic benzene hydroxylation to phenol. Appl. Catal. B.

[56] Wei D., Huang L., Liang H., Zou J., Chen W., Yang C., Hou Y., Zheng D., Zhang J. (2021). Photocatalytic hydroxylation of benzene to phenol over organosilane-functionalized FeVO_4_ nanorods. Catal. Sci. Technol..

[57] Streb C. (2012). New trends in polyoxometalate photoredox chemistry: From photosensitisation to water oxidation catalysis. Dalton Trans..

[58] Wang S.-S., Yang G.-Y. (2015). Recent Advances in Polyoxometalate-Catalyzed Reactions. Chem. Rev..

[59] Zhang L., Hou Q., Zhou Y., Wang J. (2019). Phosphotungstic anion-paired quinoline salt for heterogeneous photocatalytic hydroxylation of benzene to phenol with air. Mol. Catal..

[60] Xu P., Zhang L., Jia X., Wen H., Wang X., Yang S., Hui J. (2021). A novel heterogeneous catalyst NH_2_-MIL-88/PMo_10_V_2_ for the photocatalytic activity enhancement of benzene hydroxylation. Catal. Sci. Technol..

[61] Gu Y., Li Q., Zang D., Huang Y., Yu H., Wei Y. (2021). Light-Induced Efficient Hydroxylation of Benzene to Phenol by Quinolinium and Polyoxovanadate-Based Supramolecular Catalysts. Angew. Chem. Int. Ed..

[62] Jin X., Balasubramanian V.V., Selvan S.T., Sawant D.P., Chari M.A., Lu G.Q., Vinu A. (2009). Highly Ordered Mesoporous Carbon Nitride Nanoparticles with High Nitrogen Content: A Metal-Free Basic Catalyst. Angew. Chem. Int. Ed..

[63] García-López E., Marcì G., Bellardita M., Palmisano L., Wang X., Anpo M., Fu X. (2020). Chapter 27-Carbon nitride as photocatalyst in organic selective transformations. Current Developments in Photocatalysis and Photocatalytic Materials.

[64] Wang Y., Wang X., Antonietti M. (2012). Polymeric Graphitic Carbon Nitride as a Heterogeneous Organocatalyst: From Photochemistry to Multipurpose Catalysis to Sustainable Chemistry. Angew. Chem. Int. Ed..

[65] Samanta S., Srivastava R., Pandikumar A., Murugan C., Vinoth S. (2022). Chapter 13—Graphitic carbon nitride for organic transformation. Nanoscale Graphitic Carbon Nitride.

[66] Goettmann F., Thomas A., Antonietti M. (2007). Metal-Free Activation of CO_2_ by Mesoporous Graphitic Carbon Nitride. Angew. Chem. Int. Ed..

[67] Wang Y., Di Y., Antonietti M., Li H., Chen X., Wang X. (2010). Excellent Visible-Light Photocatalysis of Fluorinated Polymeric Carbon Nitride Solids. Chem. Mater..

[68] Chen X., Zhang J., Fu X., Antonietti M., Wang X. (2009). Fe-g-C_3_N_4_-catalyzed oxidation of benzene to phenol using hydrogen peroxide and visible light. J. Am. Chem. Soc..

[69] Zhang P., Gong Y., Li H., Chen Z., Wang Y. (2013). Selective oxidation of benzene to phenol by FeCl_3_/mpg-C_3_N_4_ hybrids. RSC Adv..

[70] Shiravand G., Badiei A., Ziarani G.M., Jafarabadi M., Hamzehloo M. (2012). Photocatalytic Synthesis of Phenol by Direct Hydroxylation of Benzene by a Modified Nanoporous Silica (LUS-1) under Sunlight. Chin. J. Catal..

[71] Ye X., Cui Y., Qiu X., Wang X. (2014). Selective oxidation of benzene to phenol by Fe-CN/TS-1 catalysts under visible light irradiation. Appl. Catal. B.

[72] Ye X., Cui Y., Wang X. (2014). Ferrocene-Modified Carbon Nitride for Direct Oxidation of Benzene to Phenol with Visible Light. ChemSusChem.

[73] Ye X., Zheng Y., Wang X. (2014). Synthesis of Ferrocene-Modified Carbon Nitride Photocatalysts by Surface Amidation Reaction for Phenol Synthesis. Chin. J. Chem..

[74] Ding Z., Chen X., Antonietti M., Wang X. (2011). Synthesis of Transition Metal-Modified Carbon Nitride Polymers for Selective Hydrocarbon Oxidation. ChemSusChem.

[75] Verma S., Nasir Baig R., Nadagouda M.N., Varma R.S. (2017). Hydroxylation of benzene via C–H activation using bimetallic CuAg@ g-C_3_N_4_. ACS Sustain. Chem. Eng..

[76] Bhuyan B., Devi M., Bora D., Dhar S.S., Newar R. (2018). Design of a Photoactive Bimetallic Cu-Au@g-C_3_N_4_ Catalyst for Visible Light Driven Hydroxylation of the Benzene Reaction through C–H Activation. Eur. J. Inorg. Chem..

[77] Hosseini S.M., Ghiaci M., Kulinich S.A., Wunderlich W., Farrokhpour H., Saraji M., Shahvar A. (2018). Au-Pd@g-C_3_N_4_ as an efficient photo-catalyst for visible-light oxidation of benzene to phenol: Experimental and mechanistic study. J. Phys. Chem. C.

[78] Basyach P., Guha A.K., Borthakur S., Kalita L., Chetia P., Sonowal K., Saikia L. (2020). Efficient hydroxylation of benzene to phenol by H_2_O_2_ using Ni-doped CuWO_4_ on carbon nitride as a catalyst under solar irradiation and its structure–activity correlation. J. Mater. Chem. A.

[79] Shao M., Cheng L., Zhang X., Ma D.D.D., Lee S.-t. (2009). Excellent Photocatalysis of HF-Treated Silicon Nanowires. J. Am. Chem. Soc..

[80] Zhu Q.-L., Xu Q. (2014). Metal–organic framework composites. Chem. Soc. Rev..

[81] Nasalevich M.A., Goesten M.G., Savenije T.J., Kapteijn F., Gascon J. (2013). Enhancing optical absorption of metal–organic frameworks for improved visible light photocatalysis. Chem. Commun..

[82] Wang D., Wang M., Li Z. (2015). Fe-based metal–organic frameworks for highly selective photocatalytic benzene hydroxylation to phenol. ACS Catal..

[83] Xu B., Chen Z., Han B., Li C. (2017). Glycol assisted synthesis of MIL-100(Fe) nanospheres for photocatalytic oxidation of benzene to phenol. Catal. Commun..

[84] Zhang L., Qiu S., Jiang G., Jiang G., Tang R. (2018). A CuII-based Metal–Organic Framework as an Efficient Photocatalyst for Direct Hydroxylation of Benzene to Phenol in Aqueous Solution. Asian J. Org. Chem..

[85] Fang Y., Zhang L., Zhao Q., Wang X., Jia X. (2019). Highly Selective Visible-Light Photocatalytic Benzene Hydroxylation to Phenol Using a New Heterogeneous Photocatalyst UiO-66-NH_2_-SA-V. Catal. Lett..

[86] Zhao Q., Zhang L., Zhao M., Xu P., Wang X., Jia X., Zhang J. (2020). Vanadium Oxyacetylacetonate Grated on Metal Organic Framework as Catalyst for the Direct Hydroxylation of Benzene to Phenol. ChemistrySelect.

[87] Titirici M.-M., White R.J., Brun N., Budarin V.L., Su D.S., del Monte F., Clark J.H., MacLachlan M.J. (2015). Sustainable carbon materials. Chem. Soc. Rev..

[88] Wang B., Anpo M., Lin J., Yang C., Zhang Y., Wang X. (2019). Direct hydroxylation of benzene to phenol on h-BCN nanosheets in the presence of FeCl_3_ and H_2_O_2_ under visible light. Catal. Today.

[89] Latorre-Sánchez M., Primo A., García H. (2013). P-doped graphene obtained by pyrolysis of modified alginate as a photocatalyst for hydrogen generation from water–methanol mixtures. Angew. Chem. Int. Ed..

[90] Xiang Q., Yu J., Jaroniec M. (2012). Graphene-based semiconductor photocatalysts. Chem. Soc. Rev..

[91] Cai J., Zhang M., Wang D., Li Z. (2018). Engineering Surface Wettability of Reduced Graphene Oxide To Realize Efficient Interfacial Photocatalytic Benzene Hydroxylation in Water. ACS Sustain. Chem. Eng..

[92] He J., Zhang M., Ana P., Hermenegildo G., Li Z. (2018). Selective Photocatalytic Benzene Hydroxylation to Phenol Using Surface-Modified Cu_2_O Supported on Graphene. J. Mater. Chem. A.

[93] Dasireddy V.D.B.C., Likozar B. (2018). Selective photocatalytic oxidation of benzene to phenol using carbon nanotube (CNT)-supported Cu and TiO_2_ heterogeneous catalysts. J. Taiwan Inst. Chem. Eng..

[94] Wang F., Chen Y., Zhu R., Sun J. (2017). Novel synthesis of magnetic, porous C/ZnFe_2_O_4_ photocatalyst with enhanced activity under visible light based on the Fenton-like reaction. Dalton Trans..

[95] Yang B., Zhang S., Gao Y., Huang L., Yang C., Hou Y., Zhang J. (2022). Unique functionalities of carbon shells coating on ZnFe_2_O_4_ for enhanced photocatalytic hydroxylation of benzene to phenol. Appl. Catal. B.

[96] Lu E., Wu J., Yang B., Yu D., Yu Z., Hou Y., Zhang J. (2020). Selective hydroxylation of benzene to phenol over Fe nanoparticles encapsulated within N-doped carbon shells. ACS Appl. Nano Mater..

[97] Asghari S., Farahmand S., Razavizadeh J.S., Ghiaci M. (2020). One-step photocatalytic benzene hydroxylation over iron (II) phthalocyanine: A new application for an old catalyst. J. Photochem. Photobiol. A.

[98] Li J., Xu Y., Ding Z., Mahadi A.H., Zhao Y., Song Y.-F. (2020). Photocatalytic selective oxidation of benzene to phenol in water over layered double hydroxide: A thermodynamic and kinetic perspective. Chem. Eng. J..

[99] Aratani Y., Oyama K., Suenobu T., Yamada Y., Fukuzumi S. (2016). Photocatalytic Hydroxylation of Benzene by Dioxygen to Phenol with a Cyano-Bridged Complex Containing FeII and RuII Incorporated in Mesoporous Silica–Alumina. Inorg. Chem..

